# Frequency of BRAF V600E immunoexpression in ameloblastomas: a multi-institutional analysis of 86 cases in Latin America and comprehensive review of the literature

**DOI:** 10.4317/medoral.26493

**Published:** 2024-04-14

**Authors:** Felipe Martins Silveira, Lauren Frenzel Schuch, Vanesa Pereira-Prado, Estefania Sicco, Oscar Almeda-Ojeda, Nelly Molina-Frechero, María Luisa Paparella, Mariana Villarroel-Dorrego, Marcela Hernadez, Ronell Bologna-Molina

**Affiliations:** 1 Department of Diagnostics in Pathology and Oral Medicine, Molecular Pathology area, School of Dentistry, University of the Repub, Uruguay; 2 Clinic of Oral Pathology, School of Dentistry, Juárez University of the State of Durango, México; 3 Health Care Department, Autonomous Metropolitan University-Xochimilco, Mexico; 4 Surgical Pathology Laboratory, Oral Pathology Department, Faculty of Dentistry, University of Buenos Aires, Argentina; 5 School of Dentistry, Central University of Venezuela, Venezuela; 6 Oral Pathology Department, School of Dentistry, University of Chile

## Abstract

**Background:**

The initiation of odontogenic tumorigenesis often involves the activation of the MAP-kinase pathway, with a pivotal role played by the BRAF V600E mutation. This study aimed to investigate the frequency of BRAF V600E immunoexpresion in ameloblastomas diagnosed in four Latin American centers and correlate this finding with the histological types and subtypes of the analyzed cases.

**Material and Methods:**

A total of 86 samples of ameloblastomas were examined for immunohistochemistry using anti-BRAF V600E antibody. The histopathological features of each case were analyzed.

**Results:**

Positivity for anti-BRAF V600E antibody was detected in 65/86 cases (75.6%). BRAF V600E was positive in 38/56 cases (67.9%) of conventional ameloblastomas and in 27/30 cases (90.0%) of unicystic ameloblastomas. A statistically significant difference in BRAF V600E positivity was observed when comparing unicystic ameloblastomas to conventional ameloblastomas (*p*=0.03). No statistically significant difference in BRAF V600E positivity was observed when comparing histological variants, both for conventional ameloblastomas and unicystic ameloblastomas.

**Conclusions:**

This study highlights a high frequency of BRAF V600E immunoreactivity in ameloblastomas among Latin American cases. The prevalence of the BRAF V600E immunoexpresion may suggest the feasibility of utilizing BRAF-targeted therapy for ameloblastomas with this mutation.

** Key words:**Immunohistochemistry, odontogenic tumors, ameloblastoma.

## Introduction

Ameloblastoma is an aggressive benign odontogenic neoplasia composed of odontogenic epithelium in a mature fibrous stroma (1,2). In the last classification of the World Health Organization/2022, ameloblastomas are classified as conventional, unicystic, peripheral, metastasizing and the recently added adenoid type. This lesion exhibits variable geographic prevalence, with a global incidence of 0.92 cases per million person-years (3). Ameloblastoma typically occurs in adults, with the average age range of diagnosis being in the third or fourth decade of life (1). The lesion commonly affects the jawbones, often causing painless swelling and alterations in facial appearance (4).

The development and causes of ameloblastoma are influenced by multiple factors, involving cellular pathways and molecular mechanisms (1,3). A deeper understanding of the condition has emerged, primarily due to the identification of the BRAF V600E mutation, which is present in around 70% of ameloblastoma cases (5). BRAF is part of the RAF family and a gene responsible for encoding B-RAF, a serine/threonine kinase protein that serves as an intermediary component in the mitogen-activated protein kinase (MAPK) signaling pathway, regulating processes such as cell proliferation and differentiation. The RAS signaling pathway initially activates RAF, which in turn triggers the MEK protein kinase. MEK then activates the ERK protein kinase. Consequently, the RAS-RAF-MEK-ERK-MAP kinase pathway acts as a communication bridge between the external environment and the cell nucleus. As BRAF plays a central role in activating MAP/ERK Kinase (MEK), any disruption of this pathway can lead to tumorigenesis. A predominant alteration within BRAF, occurring in over 90% of cases, is the BRAF V600E mutation, characterized by a substitution of valine (V) with glutamic acid (E) at codon 600. These BRAF mutations drive the activation of the MAPK pathway and the RAS/BRAF/MEK/ERK pathway becomes hyperactive, ultimately resulting in uncontrolled cell growth and the development of various human benign and malignant neoplasms, including melanotic neuroectodermal tumor of infancy, melanocytic nevi, thyroid papillary carcinoma, lung carcinoma, melanomas, and colorectal carcinomas (6,7).

Investigations into BRAF V600E mutations in ameloblastomas have been conducted in different previous studies; however, as of our current understanding, no analogous investigation specifically focused on Latin American cases has been undertaken. Considering the dynamic advancements in molecular research concerning ameloblastoma, the present study endeavors to define the prevalence of the BRAF V600E mutation in a significant sample of ameloblastomas derived from Latin American centers and to explore its potential associations with the histological variants of this odontogenic neoplasm.

## Material and Methods

- Samples

This study was approved by Ethics Committee (Faculty of Dentistry, Universidad de la República No. 091900-000184-17). Eighty-six samples of formalin-fixed paraffin-embedded (FFPE) nondecalcified ameloblastomas archived by the Research Group of the Commission for Scientific Research Sector (Comisión Sectorial de Investigación Científica, Uruguay, Research Group number 881880) were randomly retrieved from the following Latin American oral pathology services: Universidad de Chile (Chile), Universidad Central de Venezuela (Venezuela), Universidad de Buenos Aires (Argentina), Universidad Juárez del Estado de Durango (México). From each included case, the type and histological variants of ameloblastomas were defined according to the World Health Organization (WHO/2022). It was also included a sample of embryonic odontogenic tissue (tooth germ).

- Immunohistochemistry

For the immunohistochemical reactions, 3-μm sections of the ameloblastoma cases were treated with a heat retrieval solution (Reveal Decloaker, RTU; Biocare Medical) to expose the antigenic epitopes. The endogenous peroxidases were blocked with 0.9% hydrogen peroxide for 5 min each. The tissue samples were incubated with a primary antibody against BRAF V600E (BRAF V600E, Rabbit Monoclonal Antibody, Clone RM8, Bio SB, USA) for 60 min and then incubated with a biotinylated anti-mouse/anti-rabbit antibody and a streptavidin-horseradish peroxidase complex for 40 min each (mouse/rabbit ImmunoDetector Biotin Link and HRP Label; Bio SB). For the negative control samples, the primary antibody was omitted, and for positive controls, melanoma tissues were used. The reaction products were visualized using the 3,3’- diaminobenzidine-H2O2 substrate (Biocare Medical), and the sections were counterstained with Harris hematoxylin.

- Immunohistochemical analysis

The reactions were subjected to independent analysis by two oral pathologists (F.M.S., L.F.S.), and any disparities were deliberated until agreement was achieved. The presence of BRAF V600E expression was considered positive when observing staining in neoplastic epithelial cells. For the immunohistochemical analysis, BRAF V600E was classified as negative (-) or positive (+) when there was clear and unequivocal staining in a significant proportion of neoplastic cells.

- Literature review

A literature review was conducted using electronic databases, including PubMed, Web of Science, Embase, and Scopus. Laboratory studies assessing the frequency of BRAF V600E immunoexpression in ameloblastomas were considered eligible. The searches aimed to identify the ten larger-sample studies, without any time restrictions. Articles without immunohistochemistry analysis, not published in the English language or studies for which the full texts were unavailable were excluded. The search was performed using the following specific terms: (“BRAF V600E” OR BRAF-V600E) AND (ameloblastoma OR ameloblastomas). Information collected included the author's name and year of publication, the country where the study was conducted, sample size, histopathological variant, and the percentage of ameloblastomas harboring mutations in BRAF V600E.

- Statistical analysis

The association of BRAF V600E status with histopathological types and variants of the ameloblastomas was analyzed. Statistical analysis was performed using the Student's t-test because the data were independent and presented a normal distribution. The results were considered significant when *p* ≤ 0.05, and the *p value*s of significance are indicated in each Figure with an asterisk. All the analyses and graphs were performed using the GraphPad Prism 8 (San Diego, California, USA).

## Results

The absolute and relative results of the BRAF V600E immunoexpresion observed observed in this study are delineated in Table 1. Among the 86 cases of ameloblastomas analyzed in this study, 56 composed the conventional type, and 30 the unicystic type. The histological subtypes of conventional ameloblastomas included the following: follicular (42 / 75.0%), desmoplastic (7 /12.5%), plexiforme (6/10.7%), and acanthomatous (1/1.8%); among the unicystic ameloblastomas, the variants observed were: luminal (16/53.3%), mural (9/30.0%), and intraluminal (5/16.7%).

- BRAF V600E immunoexpression

**Figure 1:**
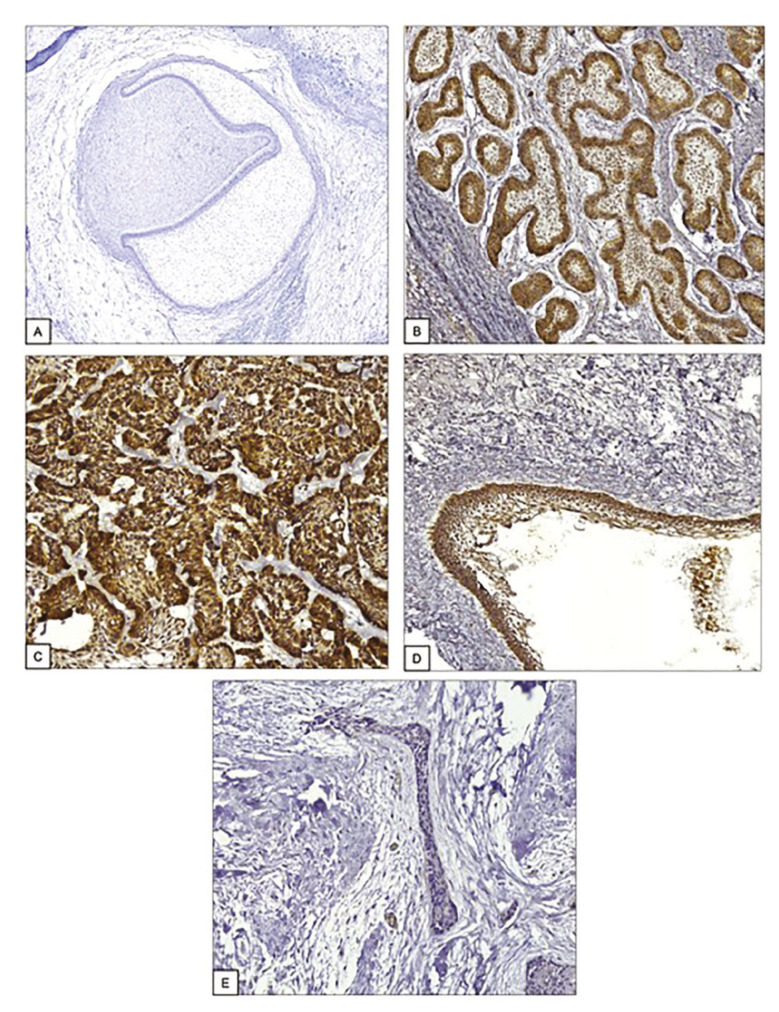
Immunohistochemical expression of BRAF V600E in dental germ and ameloblastomas. A, dental germ (9x). B, conventional ameloblastoma, follicular type (20x). C, conventional ameloblastoma, plexiform type (20x). D, unicystic ameloblastoma (20x). E, desmoplastic ameloblastoma (20x).

The embryonic odontogenic tissue was negative for BRAF V600E immunoexpression. The positivity for BRAF V600E in this sample of ameloblastomas was predominantly observed in the odontogenic epithelial cells. In the conventional ameloblastomas, positive cases exhibited positivity in the peripheral basal cells and in central cells resembling the stellate reticulum. In the unicystic ameloblastomas, it was observed positivity in the cystic epithelium and in the tumor islands infiltrating the fibrous wall in specific cases. It was not observed positivity in the stromal tissue (Fig. 1).

**Figure 2:**
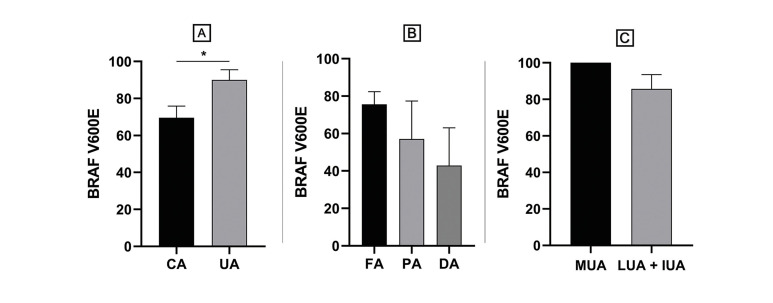
Immunohistochemical expression of BRAF V600E was compared between conventional ameloblastomas and unicystic ameloblastomas (A) and between histological subtypes of conventional ameloblastomas (B) and unicystic ameloblastomas (C). Statistically significant differences were found only in the comparison between conventional ameloblastomas and unicystic ameloblastomas (*p=0.03) (Student’s t test). CA, conventional ameloblastoma; UA, unicystic ameloblastoma; FA, follicular ameloblastoma; PA, plexiforme ameloblastoma; DA, desmoplastic ameloblastoma; MUA, mural unicystic ameloblastoma; LUA, luminal unicystic ameloblastoma; IUA, intralumninal unicystic ameloblastoma.

From the total of cases analyzed (*n*=86), 65 cases (75.6%) demonstrated positivity for BRAF V600E. Regarding conventional ameloblastomas (*n*=56), 38 cases (67.9%) were positive for BRAF V600E. Among the unicystic ameloblastomas (*n*=30), 27 cases (90.0%) were positive for BRAF V600E. A statistically significant difference in BRAF V600E positivity was observed when comparing unicystic ameloblastomas to conventional ameloblastomas (*p*=0.03) (Fig. 2). Within the conventional ameloblastomas, histological subtyping revealed the following for BRAF V600E immunoexpression: 32 cases (76.2%) of follicular ameloblastomas were positive; 3 cases (42.9%) of desmoplastic ameloblastomas were positive; and, for the plexiforme ameloblastomas, 3 cases (50.0%) were positive and 3 cases (50.0%) were negative. There was no statistically significant difference in BRAF V600E positivity when comparing the histological variants of conventional ameloblastomas (Fig. 2). The only case of acanthomatous ameloblastoma included in the study was negative. Within the unicystic ameloblastomas, all luminal and mural variants tested positive, while 60% of intraluminal variants were negative for BRAF V600E immunoexpression. For the statistical analysis of the histological subtypes of unicystic ameloblastomas, intraluminal and luminal unicystic ameloblastomas were considered a single group. It was not observed a statistical significant difference between mural unicystic ameloblastomas and luminal + intraluminal unicystic ameloblastomas (*p*=0.246) (Fig. 2).

- Comprehensive literature review

The results of the literature review performed in this study are delineated in Table 2. The literature review on the four databases yielded 264 results, from which the 10 largest case series investigating BRAF V600E mutation through immunohistochemistry in ameloblastomas were selected (8-17). Among the three largest case series, two were conducted in Brazil, with 128 and 84 cases, respectively. The remaining studies were performed in the United States (3 studies), Finland (1 study), Thailand (2 studies), Iran (1 study), and Pakistan (1 study). Of the 10 included series, a total of 691 cases were examined, from which positive immunoexpression for BRAF V600E ranged from 61.3% to 84%. Five studies differentiated the immunohistochemical expressions of BRAF V600E between conventional and unicystic ameloblastomas, of which three (60%) demonstrated that the unicystic types exhibited higher positivity compared to the conventional ones.

## Discussion

The study of the pathogenesis involved in the development of ameloblastomas is of paramount importance for understanding the molecular aspects of this type of odontogenic tumor. Recent studies have provided robust evidence that the activation of the MAPK signaling pathway plays a significant role in the pathogenesis of this odontogenic tumor, with SMO and BRAF V600E mutations being the most observed (18). In this context, this is the first multi-institutional study evaluating the landscape related to the BRAF V600E mutation in a substantial sample of ameloblastomas diagnosed in Latin America. Also, to the best of our knowledge, our study represents the second largest series evaluating BRAF V600E mutation in ameloblastomas specifically derived from Latin American services. Through immunohistochemical analysis, this study revealed a substantial percentage of 75.6% of ameloblastomas harboring mutations in BRAF V600E, with the highest significance observed in unicystic cases, where 90% of them tested positive for this mutation.

Different studies have reported mutations in BRAF V600E in ameloblastomas. According to a recent systematic review and meta-analysis on this topic, mutation rates for BRAF V600E in ameloblastomas range from 33.33% to 92.0% (19); and according to the largest case series reviewed in this study, of the 691 cases evaluated, the immunohistochemical positivity for BRAF V600E ranged from 61.3% to 84% (Table 2). In addition, the meta-analysis of Yusof *et al*. (19) reported a mutation prevalence of 70.49% in the BRAF V600E gene in patients with ameloblastoma, a rate very similar to that reported in the present study. Other significant studies from Latin American countries have assessed the frequency of BRAF V600E mutation in ameloblastomas by immunohistochemical analysis: Fregnani *et al*. (20) reported a frequency of 46.6% from 73 cases; Canto *et al*. (9) reported a frequency of 78.6% from 84 cases; Santana *et al*. (21) reported a frequency of 66.7% from 30 cases; and Marcelino *et al*. (8) reported a frequency of 81.2% from 128 cases. It should be noted that the study of Marcelino *et al*. (8) also used DNA sequencing for BRAF V600E mutation in association with the immunohistochemistry. These results demonstrate that, despite a wide variation in the frequencies of ameloblastomas harboring BRAF V600E mutations are observed in different studies, the overall prevalence of this mutation is relatively high in Latin American samples.

In this series of Latin American ameloblastomas, the positivity for BRAF V600E in unicystic types was statistically higher when compared to conventional types. In this context, according to the largest case series reviewed in this study that compared the immunoexpression of BRAF V600E in conventional and unicystic ameloblastomas, there was also a trend indicating that unicystic types exhibit higher positivity for BRAF V600E than conventional types (8,11,13). Comparatively, in the study by Togni *et al*. (22), a statistical association between BRAF V600E and unicystic ameloblastomas was also demonstrated. However, in these cases, the association was also linked to the location of the cases in the mandible and the use of other molecular techniques in addition to immunohistochemistry. Unicystic ameloblastoma is a distinguishable entity of ameloblastomas, characterized by slow growth and sometimes defined as a less aggressive type (23). These results may suggest that, despite clinicopathological and prognostic differences between conventional and unicystic ameloblastomas, the BRAF V600E mutation could be a significant event also in the pathogenesis of unicystic ameloblastomas. The mutation of BRAF V600E may be present in ameloblastomas regardless of differences in prognostic and clinicopathological factors.

In this study, potential relationships between the histological subtypes of ameloblastomas and BRAF V600E mutation were evaluated. In conventional ameloblastomas, no statistical association was observed between any of their histological variants and BRAF V600E positivity. Other significant studies on this subject could also not correlate histological subtypes with the presence of BRAF V600E (20,24,25). It is important to note that the case of acanthomatous ameloblastoma was not included in the statistical analysis of this study, as it was a solitary case. On the other hand, in Sweeney's traditional study (18), a correlation is demonstrated with the histological pattern of ameloblastomas, where most cases of follicular or desmoplastic ameloblastomas exhibited mutations in either SMO or BRAF genes. For the unicystic ameloblastomas, in the present study, it was observed positive immunoexpression of BRAF V600E for either luminal, intraluminal and mural subtypes, confirming that these three histological subtypes may be positive for BRAF V600E mutation, as previously defined (11). The three distinct subtypes of unicystic ameloblastoma are based on the ameloblastomatous epithelium's proliferation patterns and there is consensus that the two subtypes luminal and intraluminal may be managed conservatively, while questions persist regarding whether the mural subtype should be regarded as a form of conventional ameloblastoma (2). In light of these potential distinctions among the histological subtypes of unicystic ameloblastomas, we combined the results of the luminal and intraluminal types to compare the immunohistochemical expression of BRAF V600E with the mural types. Our analysis did not reveal a statistically significant difference between them, what may suggests that there are no molecular differences regarding BRAF V600E among these subtypes. Therefore, these findings may suggest that BRAF mutations do not correspond to specific histologic subtypes of ameloblastoma. However, further studies employing additional molecular techniques are necessary to corroborate this finding.

In the present study, we exclusively employed immunohistochemistry as the technique for BRAF V600E (clone RM8) mutation analysis. While molecular tests are considered the gold standard for gene mutation detection in several types of solid tumors, its cost and limited availability hindered the assessment of BRAF V600E mutations (19,26). In the study of Marcelino *et al*. (8), the authors identified 81.2% as positive and 18.8% as negative ameloblastomas for the anti-BRAF V600E antibody by immunohistochemistry; and, for PCR analysis, it was showed 82.8% cases of ameloblastomas as positive and 17.2% as negative for the BRAF V600E mutation. Mendez *et al*. (27) analyzed 46 ameloblastomas and demonstrated that, among mandibular ameloblastomas, 83.8% of the cases showed positive BRAF VE1 by immunohistochemistry, with no positive cases in maxillary ameloblastomas. When comparing with allele-specific PCR, 82.6% of mandibular ameloblastomas and 0% of maxillary ameloblastomas had the BRAFV600E mutation. In addition, all BRAF-wild type ameloblastomas also tested negative by immunohistochemistry. These studies confirm that BRAF V600E immunopositivity is correlated with BRAF V600E mutation status in ameloblastomas and can be considered a good method used as an initial screening test in identifying this mutation in ameloblastomas due to its simplicity, reliability, cost-effectiveness, and widespread adoption as the standard histopathological diagnostic procedure. Furthermore, it is crucial to emphasize that the immunohistochemical technique targeting BRAF V600 can provide valuable assistance in the differential diagnosis of ameloblastomas, particularly when distinguishing them from cystic lesions. This is especially relevant in cases involving incisional biopsies or situations of severe inflammation where the histological pattern of the lesion may be altered (28).

On the other hand, it is also crucial to highlight that different studies have demonstrated discrepancies between immunohistochemistry and molecular testing results for BRAF V600E, with false positives or false negatives in immunohistochemical techniques across different odontogenic lesions. Additionally, a study revealed discrepancies even among different antibodies (VE1) used in immunohistochemistry, underscoring the importance of employing molecular tests to determine the presence of BRAF V600E mutations (29). In this context, in the study of Togni *et al*. (22), immunohistochemistry, Sanger sequencing, and Matrix-Assisted Laser Desorption/Ionization-Time of Flight mass spectrometry (Sequenom) were conducted to evaluate the BRAFV600E mutation in odontogenic lesions, revealing that 3 and 1 cases of BRAF wild-type (as determined by immunohistochemistry and Sanger sequencing, respectively) yielded BRAF-positive results in the Sequenom analysis. This study demonstrated a sensitivity of 100% and specificity of 98.1% through Sequenom, confirming it as a highly sensitive and specific technology for detecting genetic variations. While Sanger sequencing and Sequenom precisely determine the mutation and serve as confirmatory methods for immunohistochemical results, as mentioned earlier, they are more complex techniques and are not as readily available as immunohistochemistry in different oral pathology services.

The results presented in this study may represent another reference supporting the use of targeted therapies for MAPK pathway mutations mainly BRAF V600E for the treatment of ameloblastomas. The primary treatment option for ameloblastomas is complete surgical resection, which results in the complete eradication of neoplasia in most cases. The identification of activated molecular pathways hints at innovative molecular-targeted treatment options for ameloblastoma, with the potential to mitigate surgical complications in cases involving resection, recurrent ameloblastoma, and metastatic ameloblastoma, although its role is still poorly defined. Promising candidates for molecular targeted therapy in ameloblastoma include vemurafenib and dabrafenib to target mutated BRAF, trametinib for MEK mutations, and ponatinib and regorafenib for FGFR2 mutations; vemurafenib and dabrafenib for BRAF and trametinib for MEK were three molecular targeted therapies for BRAF V600E mutations sanctioned by the US Food and Drug Administration (30).

It is important to highlight some limitations of the present study. While immunohistochemistry is indicated for assessing the presence of BRAF V600E, it is essential to emphasize again that in this study the results are solely based on immunohistochemical expression. Additionally, clinical factors of the studied ameloblastomas that could influence the expression of BRAF V600E, such as tumor location, were not taken into account, and it could be a factor influencing the studied cases in this sample. It has been demonstrated that there is a predominance of mandibular and younger occurring ameloblastomas exhibiting BRAF mutations and some studies have shown an association between this mutation and tumor aggressiveness (18).

## Conclusions

The present study reveals a high frequency of ameloblastomas displaying positive immunoreactivity for BRAF V600E in a significant sample of Latin American cases. These results further validate the important role of this mutation in both conventional and unicystic ameloblastomas, regardless of their histological subtype. It is imperative to delve deeper into research by assessing the outcomes of targeted therapies for BRAF inhibition in cases of mutated ameloblastomas.

## Figures and Tables

**Table 1:** Immunoexpression for BRAF V600E according to histological subtypes in ameloblastomas from Latin American services.

Ameloblastoma		BRAF V600E		
		Negative n (%)	Positive n (%)	Total n (%)
Conventional	Follicular	10 (23.8)	32 (76.2)	42 (75.0)
	Desmoplastic	4 (57.1)	3 (42.9)	7 (12.5)
	Plexiforme	3 (50.0)	3 (50.0)	6 (10.7)
	Acanthomatous	1 (100.0)	-	1 (1.8)
	Total	18 (32.1)	38 (67.9)	56 (100.0)
Unicystic	Luminal	-	16 (100.0)	16 (53.3)
	Mural	-	9 (100.0)	9 (30.0)
	Intraluminal	3 (60.0)	2 (40.0)	5 (16.7)
	Total	3 (10.0)	27 (90.0)	30 (100.0)
Total		21 (24.4)	65 (75.6)	86 (100.0)

**Table 2:** Positive immunoexpression for BRAF V600E in ameloblastomas in the 10 largest published series.

Author, year	Country	Sample size	BRAF V600E
Marcelino et al., 2021 (8)	Brazil	128	81.2
		Conventional (n=110)	80.9
		Unicystic (n=18)	83.3
Do Canto et al., 2019 (9)	Brazil	84	78.6
		Conventional (n=73)	79.4
		Unicystic (n=11)	72.3
Brown et al., 2014 (10)	USA	84	64.2
Heikinheimo et al., 2019 (11)	Finland	69	72.4
		Conventional (n=31)	61.3
		Unicystic (n=38)	81.6
Kunmongkolwut et al., 2022 (12)	Thailand	74	67.6
Lapthanasupkul et al., 2020 (13)	Thailand	73	79.4
		Conventional ( n=51)	72.5
		Unicystic (n=22)	95.5
Derakhshan et al., 2020 (14)	Iran	50	84
Mendez et al., 2022 (15)	USA	46	67.4
Owosho et al., 2021 (16)	USA	44	67.3
Bashir et al., 2022 (17)	Pakistan	39	64.1
		Conventional (n=30)	90
		Unicystic (n=8)	25
		Peripheral (n=1)	0
